# Improved Access to Organo‐Soluble Di‐ and Tetrafluoridochlorate(I)/(III) Salts

**DOI:** 10.1002/anie.202006268

**Published:** 2020-07-15

**Authors:** P. Pröhm, J. R. Schmid, K. Sonnenberg, P. Voßnacker, S. Steinhauer, C. J. Schattenberg, R. Müller, M. Kaupp, S. Riedel

**Affiliations:** ^1^ Freie Universität Berlin Institut of Chemistry and Biochemistry Fabeckstr. 34/36 14195 Berlin Germany; ^2^ Technische Universität Berlin Department of Chemistry: Theoretical Chemistry Sekr. C7, Strasse des 17. Juni 135 10623 Berlin Germany

**Keywords:** chlorine fluorides, fluorination reagents, strong oxidizers

## Abstract

A facile one‐pot gram‐scale synthesis of tetraalkylammonium tetrafluoridochlorate(III) [cat][ClF_4_] ([cat]=[NEt_3_Me]^+^, [NEt_4_]^+^) is described. An acetonitrile solution of the corresponding alkylammonium chloride salt is fluorinated with diluted fluorine at low temperatures. The reaction proceeds via the [ClF_2_]^−^ anion which is structurally characterized for the first time. The potential application of [ClF_4_]^−^ salts as fluorinating agents is evaluated by the reaction with diphenyl disulfide, Ph_2_S_2_, to pentafluorosulfanyl benzene, PhSF_5_. The CN moieties in acetonitrile and [B(CN)_4_]^−^ are transferred in CF_3_ groups. Exposure of carbon monoxide, CO, leads to the formation of carbonyl fluoride, COF_2_, and elemental gold is dissolved under the formation of tetrafluoridoaurate [AuF_4_]^−^.

## Graphical abstract

**The synthesis of** tetraalkylammonium tetrafluoridochlorate(III) is described. An acetonitrile solution of the alkylammonium chloride salt is reacted with diluted fluorine at low temperature. The application as fluorinating agent is evaluated by the reaction with Ph_2_S_2_ to PhSF_5_, the transformation of the CN moieties in acetonitrile and [B(CN)_4_]^−^ into CF_3_, reaction with CO to COF_2_ and the formation of tetrafluoridoaurate [AuF_4_]^−^ with elemental gold.

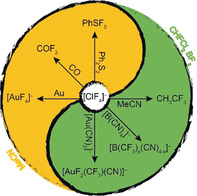

Chlorine fluorides (ClF, ClF_3_, ClF_5_) are amongst the most reactive compounds known.^1^ These very strongly oxidizing gases should only be handled in special equipment made from metal, including stainless steel, copper, nickel, Monel and other Cu/Ni alloys or from perfluorinated polymers such as PTFE, KEL‐F or PFA. Especially ClF_3_ and ClF_5_ can under certain conditions exceed the reactivity of elemental fluorine. Exposure to organic material leads to violent reactions in many cases and only carefully chosen reaction conditions (especially dilution of the chlorine fluoride) can avoid dangerous explosions; however, acetonitrile is known to be resistant against bromo and chloro fluorine compounds.^2^ ClF_3_ reacts with nitrosyl fluoride or alkali metal fluorides under formation of the corresponding tetrafluoridochlorate(III) salts.^3^, ^4^ Alkali metal tetrafluoridochlorates can also be formed via exposure of the corresponding alkali metal chlorides (CsCl, RbCl, KCl) towards elemental fluorine at elevated temperatures.^5^ More soluble tetrafluoridochlorate(III) salts, for example, alkylammonium salts, can be obtained by salt metathesis with the corresponding alkylammonium fluorides in propionitrile at low temperatures.^3^ However, only few examples of stable anhydrous alkylammonium fluorides are known.^6^ Nevertheless, cation metathesis was used to synthesize the tetramethylammonium and *1,1,3,3,5,5*‐hexamethylpiperidinium (pip) tetrafluoridochlorate(III) salts.^3^, ^7^ Additionally, [NMe_4_][ClF_4_] was observed as a decomposition product of [NMe_4_][ClF_6_].^8^ The difluoridochlorate(I) anion, [ClF_2_]^−^, is so‐far only reported with a limited amount of counter ions (K^+^, Rb^+^, Cs^+^, NO^+^) and only characterized by vibrational spectroscopy.^9^, ^10^ To the best of our knowledge the chemistry of di‐ and tetrafluoridochlorates was only studied rudimentarily. Hence, we present a ClF_3_‐free, gram scale synthesis of organo‐soluble tetraalkylammonium tetrafluoridochlorate and explore its chemical properties. We exposed triethylmethylammonium chloride [NEt_3_Me]Cl to dilute fluorine (10 % in argon) in acetonitrile or propionitrile at low temperatures [Equation (1)]. In the beginning of the fluorination a slight yellow color of the solution was observed. We hypothesize that small amounts of chlorine are formed. However, it was not possible to detect any vibrational band of Cl_2_ via Raman spectroscopy. In the process of further fluorination the solution decolorized again. For the synthesis of highly concentrated solutions we used [NEt_3_Me][Cl_3_]^11^ as a starting material due to its enhanced solubility in acetonitrile in comparison to tertraalkylammonium chlorides like [NEt_4_]Cl or [NMe_4_]Cl. It is worth mentioning that all starting materials are commercially available and the reaction proceeds in standard laboratory glassware in contrast to the reported synthesis with ClF_3_.

(1) $$\left[\mathrm{NEt}{}_{3}\mathrm{Me}\right]\mathrm{Cl}+2\;F{}_{2}\overset{\mathrm{RCN}}{\underset{-35\;{}^{\circ }C,\;R=\mathrm{Me},\mathrm{Et}}{\to }}\left[\mathrm{NEt}{}_{3}\mathrm{Me}\right]\left[\mathrm{ClF}{}_{4}\right]$$

We characterized the obtained solution by Raman and ^19^F NMR spectroscopy. The ^19^F NMR spectrum (Figure S2 in the Supporting Information) shows one main resonance at 67 ppm for [ClF_4_]^−^ which is in good agreement with previously reported values (66.8 ppm).^7^ The Raman spectrum (Figure 1) measured at −196 °C shows, besides the bands of the cation and solvent, three bands at 500 cm^−1^, 408 cm^−1^ and 278 cm^−1^, which are attributed to the a_1g_, the b_1g_, and the b_2g_ vibration in the *D*_4*h*_ symmetric molecule in agreement with literature values (508 cm^−1^, 415 cm^−1^, 278 cm^−1^).^7^, ^12^

**Figure 1:**
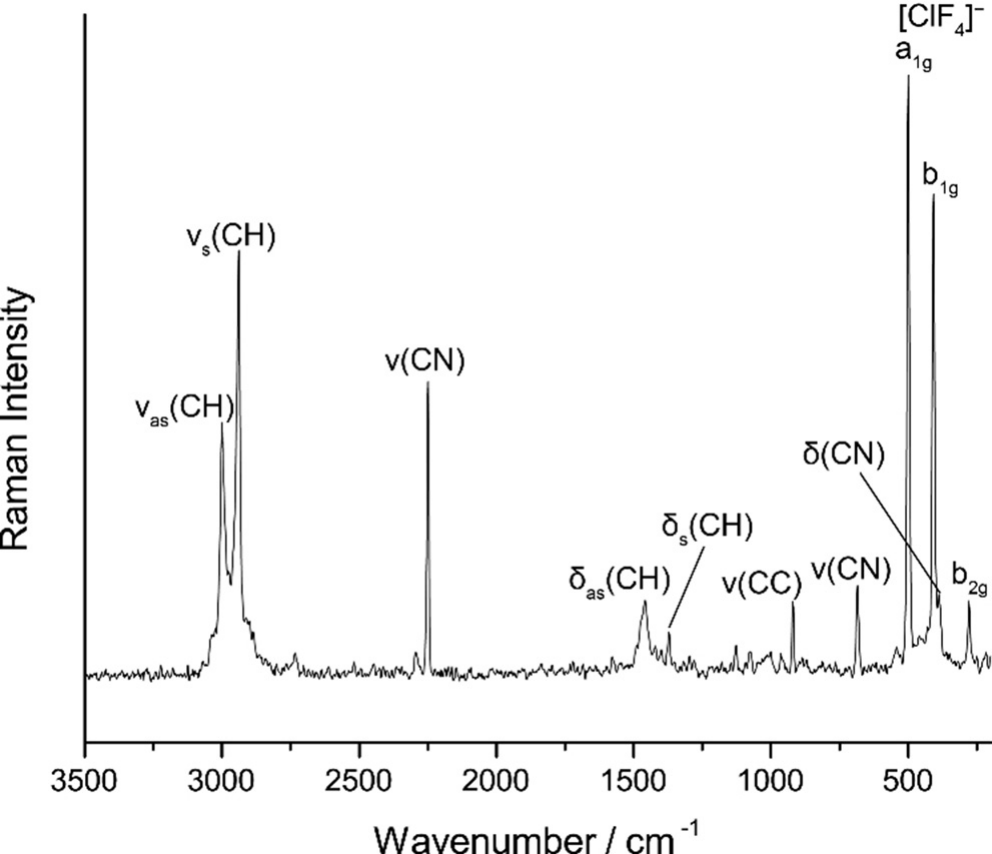
Raman spectrum of [NEt_3_Me][ClF_4_] in acetonitrile at −196 °C.

By exchange of the cation to tetraethylammonium [NEt_4_]^+^ we were able to grow single crystals suitable for single crystal X‐Ray diffraction. [NEt_4_][ClF_4_] crystallizes in the space group *C*2/*c*. The chlorine atom occupies the Wyckhoff position 4*c* (site symmetry *P* $\bar{1}$ ). The [ClF_4_]^−^ is only slightly distorted from *D*_4*h*_ symmetry with two crystallographically inequivalent Cl−F bonds, *d*(Cl−F1)=180.6(2) pm and *d*(Cl−F2)=179.3(2) pm and rectangular bond angles ∡(F1‐Cl‐F2)=90.01(5)° and ∡(F2‐Cl‐F1′)=89.99(5)°, see Figure 2. Overall, the anion is in good agreement with the structures reported in the literature ([cat][ClF_4_], [cat]=K^+^, Rb^+^, Cs^+^, NO^+^_,_ [pip]^+^) and is only slightly less distorted than in the reported structures of [pip][ClF_4_] and [NO][ClF_4_] (see Table S2).^3^ The shortest cation anion contact is an F−H hydrogen bridge and was determined to 242.3(1) pm, the corresponding F⋅⋅⋅(H)−C distance was determined to 336.5(3) pm. Additionally, we calculated the Hirshfeld surface which is also showing a short cation anion contact (Figure S16).

**Figure 2:**
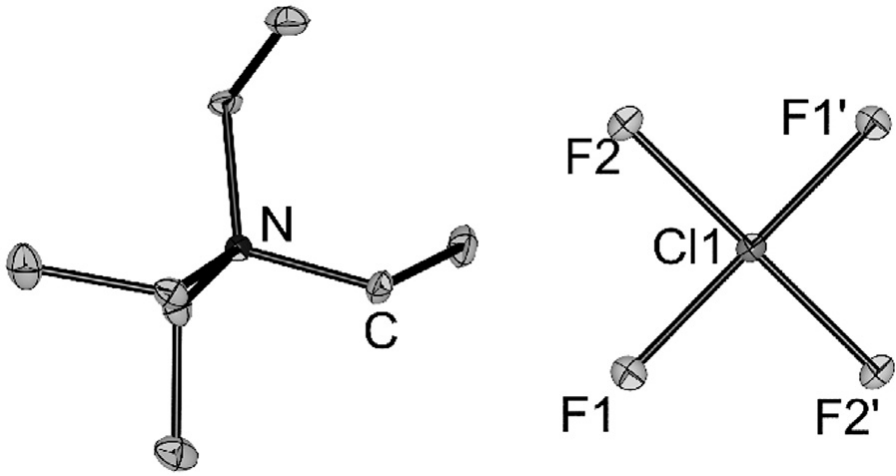
Crystal structure [NEt_4_][ClF_4_]. Displacement ellipsoids are shown at 50 % probability at 100 K. Selected bond lengths [pm] and bond angles [°]: F1–Cl1 179.2(2), F2–Cl1 180.6(2) F1‐Cl1‐F2 89.99(5), F2‐Cl1‐F1′ 90.01(5). Hydrogen atoms omitted for clarity.^29^

The addition of 1.2 equiv. fluorine to a solution of [NEt_3_Me]Cl in acetonitrile yields a mixture of [ClF_2_]^−^ and [ClF_4_]^−^ anions. Again, we were able to characterize this mixture by vibrational and ^19^F NMR spectroscopy. The Raman spectrum (Figure 3) of this mixture at −196 °C shows the characteristic bands of [ClF_4_]^−^ and additionally one band at 455 cm^−1^ which can be assigned to the symmetric stretch vibration of [ClF_2_]^−^, similar to those reported for solid KClF_2_ (475 cm^−1^) and RbClF_2_ (476 cm^−1^).^10^ The harmonic frequency of the symmetric stretching mode for free [ClF_2_]^−^ is calculated to 453 cm^−1^ (CCSD(T)/def2‐TZVPP) which is in good agreement with our assignment.^13^ The differences between the value reported by us and the literature values can be explained by the stronger coordination of the alkali metal cation in the solid‐state in comparison with the tetraalkylammonium cation.

**Figure 3:**
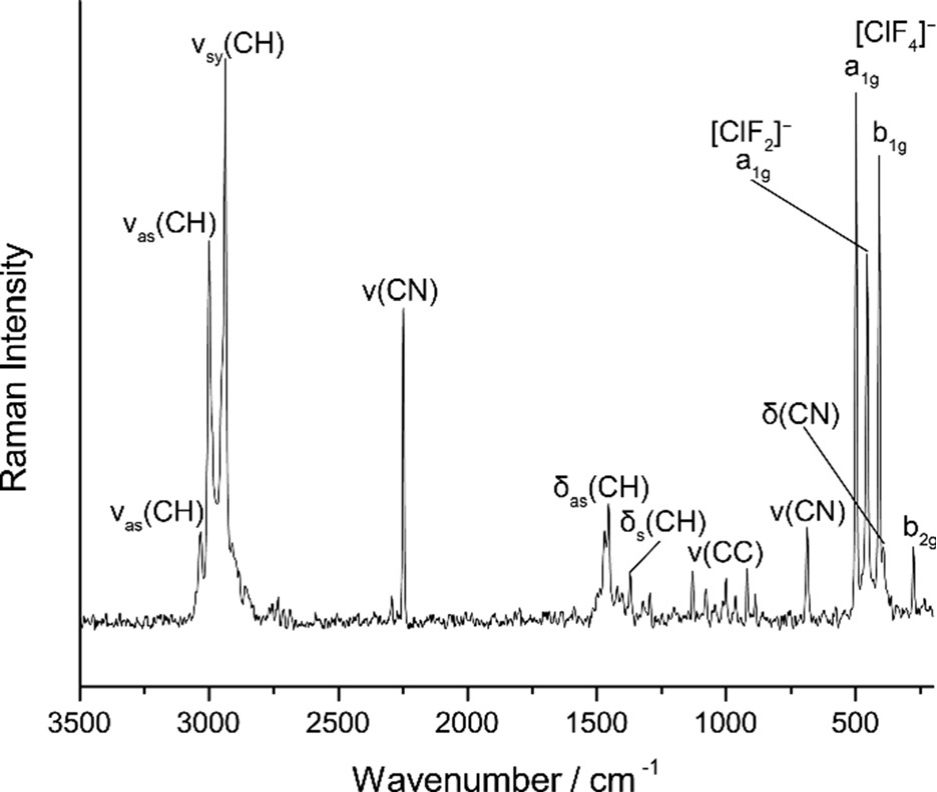
Raman spectrum of [NEt_3_Me]_3_[ClF_4_][ClF_2_]_2_ in acetonitrile at −196 °C.

The ^19^F NMR spectrum (Figure S3) shows two signals. The one at 67 ppm can be assigned to the [ClF_4_]^−^ anion (see above). We assigned the second signal at −125 ppm to the [ClF_2_]^−^ anion since the Raman spectrum showed the corresponding band at 455 cm^−1^ before and after the measurement of the NMR spectrum.

Table 1 provides our computed ^19^F shifts for the full series of anions [XF_*n*_]^−^ (X=Cl, Br, I; *n*=2, 4, 6), using two functionals (the BHLYP global hybrid and the LH12ct‐SsifPW92 local hybrid) that have been shown to provide superior ^19^F shieldings compared to B3LYP.^14^, ^15^ The computations used BP86‐D3(BJ)(COSMO,CH_3_CN))/def2‐TZVPPD structures and DFT(COSMO,CH_3_CN)‐GIAO//pcSseg‐4/ANO‐RCC‐unc shielding computations (with pcSseg‐4 basis sets for F, Cl, Br and the uncontracted ANO‐RCC basis for I; see Supporting Information for further computational details and additional data). The available experimental data for *n*=4, 6 are reproduced rather well at the two levels used. As shown by separate four‐component relativistic computations (Table S5 in Supporting Information), both spin‐orbit and scalar relativistic effects are small, in most cases a few ppm, at most about 13 ppm for [IF_2_]^−^. The small spin‐orbit effects can be understood from an inefficient transfer mechanism to the fluoride nuclei.^16^ However, the computed ^19^F shifts for the difluoridohalide anions are too shielded by 60–100 ppm (more so for BHLYP than for LH12ct‐SsifPW92). This is clearly outside the error margins of these two density functionals or of relativistic contributions. Closer inspection reveals that the highly negative fluorine charges in the difluorido anions give rise to specific F⋅⋅⋅H−C interactions with the acetonitrile solvent, which is not the case for the four‐ and six‐coordinate cases. These strong interactions are not covered by the implicit COSMO solvent model but become apparent when using more explicit treatments of solvation. Detailed studies of these interesting fluoro‐specific interactions are underway and will be reported elsewhere.

**Table 1:** Calculated ^19^F NMR chemical shifts relative to CFCl_3_ (in ppm) of [XF_*n*_]^−^ (X=Cl, Br, I; *n*=2, 4, 6) in comparison with experimental values.

Molecule	*δ*_exp_	*δ*_BHLYP_^[a]^	*δ*_LH12ct‐sifPW92_^[a]^
[ClF_2_]^−^	−125^[b]^	−202	−174
[ClF_4_]^−^	67^7^	68	61
[ClF_6_]^−^	–	278	249
[BrF_2_]^−^	−2103a	−296	−273
[BrF_4_]^−^	−37^8^	−42	−45
[BrF_6_]^−^	94^8^	129	112
[IF_2_]^−^	−286, −2823a, 17	−360	−348
[IF_4_]^−^	−1063a, 18	−111	−117
[IF_6_]^−^	13^19^	30	14

[a] DFT(COSMO,CH_3_CN)‐GIAO/pcSseg‐4/ANO‐RCC‐unc//BP86‐D3(BJ)(COSMO,CH_3_CN))/def2‐TZVPPD data. [b] This work.

By slowly cooling a reaction mixture of [NEt_3_Me]Cl with 1.2 equiv. fluorine in acetonitrile, single crystals of [NEt_3_Me]_3_[ClF_4_][ClF_2_]_2_ were obtained (for further details see Supporting Information, Figure S17). Replacing F_2_ by ClF as a fluorination agent we were able to synthesize neat [NEt_3_Me][ClF_2_]. The Raman spectrum (Figure S6) shows one main band at 457 cm^−1^ which is in good agreement with data from [NEt_3_Me]_3_[ClF_4_][ClF_2_]_2_ (455 cm^−1^). The compound crystallizes in the space group *P*12_1_/*c*1 (Figure 4). The two anionic moieties F1Cl1F1′ and F2ACl2F2A′ are both half occupied with Cl1 on Wyckhoff position 2b (site symmetry *P* $\bar{1}$ ) and Cl2 on Wyckhoff position 2c (site symmetry *P* $\bar{1}$ ). The Cl−F bond lengths are 185.24(6) pm (Cl1−F1) and 184.6(2) (Cl2−F2A). The calculated bond length of the free [ClF_2_]^−^ is 186.8 pm (CCSD(T)/def2‐TZVPP).^13^ The bonding situation is best described by a 3‐center‐4‐electron bond. The Cl−F bond lengths in [ClF_2_]^−^ are significantly elongated in comparison with solid ClF (162.8(1) pm).^20^ It is well‐known from polyhalide chemistry that the bond length of a dihalogen is elongated upon coordination by a Lewis base due to the donation of electron density into the σ*(Cl−F) orbital.^12^, ^21^ As anticipated, the [ClF_2_]^−^ anion is computed to be more thermochemically stable towards halogen loss than [Cl_3_]^−^ and [F(Cl)_2_]^−^ which is due to more ionic interactions of the ClF moiety in comparison to Cl_2_.^12^, ^22^

**Figure 4:**
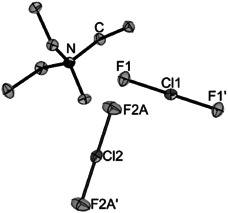
Crystal structure of [NEt_3_Me][ClF_2_]. Displacement ellipsoids are shown at 50 % probability at 100 K. Selected bond lengths [pm]: F1–Cl1 185.24(6), F2A–Cl2 184.6(2). Hydrogen atoms omitted for clarity.^29^

We examined the stability of [NEt_3_Me][ClF_4_] in acetonitrile solution via ^19^F NMR spectroscopy. Surprisingly, it showed only slow decomposition at room temperature over a month via fluorination of the organic solvent and the cation. However, the isolated solid is significantly more reactive. We observed explosions in several cases at temperatures above −40 °C. Consequently, we avoided the isolation of larger quantities of [NEt_3_Me][ClF_4_] and instead worked with solutions in propionitrile or acetonitrile with concentrations in the range of 0.66 mol l^−1^ to 8.8 mol l^−1^.

We envisioned [NEt_3_Me][ClF_4_] as a fluorinating and oxidation reagent for the synthesis of highly fluorinated moieties such as trifluoromethyl (‐CF_3_), pentafluorosulfanyl (‐SF_5_) or fluoridometallates ([MF_*x*_]^−^). Trifluoromethyl and pentafluorosulfanyl derivatives have a growing importance in pharmaceutical‐ and agrochemistry.^23^, ^24^, ^25^ Recently, Togni and co‐workers reported on a non‐gaseous reagent to access aryl tetrafluorido‐λ^6^‐sulfanyl chlorides (Ar‐SF_4_Cl), a key intermediate for the synthesis of pentafluorosulfanyl aryls (Ar‐SF_5_).^24^ Beier and co‐workers recently studied the direct fluorination with dilute elemental fluorine and disulfides to directly obtain pentafluorosulfanyl aryls, a process with industrial application.^25^ As a proof of concept we exposed diphenyl disulfide, Ph_2_S_2_, to a solution of [NEt_3_Me][ClF_4_] in propionitrile at −50 °C to directly obtain phenylsulfur pentafluoride, PhSF_5_. As by‐products the *cis*‐ and *trans*‐PhSF_4_Cl were observed (Table 2 Entry 1, for further details see Supporting Information). PhSF_5_ can be prepared in pure form by removal of both PhSF_4_Cl isomers by hydrolysis. Simultaneously, residual fluorochlorates are hydrolysed.^26^

**Table 2:** Reactivity Studies with [NEt_3_Me][ClF_4_].

Entry	Substrate	Lewis acid	Product
1	Ph_2_S_2_	–	PhSF_5_, PhSF_4_Cl
2	MeCN	BF_3_	MeCF_3_
3	[B(CN)_4_]^−^	BF_3_	[B(CF_3_)_*x*_(CN)_4−*x*_]^−^
4	[Au(CN)_2_]^−^	BF_3_	*cis*‐[AuF_2_(CF_3_)(CN)]^−[a]^
5	Au	–	[AuF_4_]^−^
6	CO	–	COF_2_

[a] Minor product.

Addition of the Lewis acid boron trifluoride, BF_3_, to a solution of [NEt_3_Me][ClF_4_] in acetonitrile led to the formation of CH_3_CF_3_, the CN activation product of the solvent acetonitrile, amongst other components. To increase the selectivity of this reaction, we synthesized the acetonitrile‐BF_3_ complex and substituted the solvent, from nitrile‐based solvents to chlorofluorocarbons. Dichlorofluoromethane (CHFCl_2_, R‐21) turns out to be sufficiently stable towards F_2_ and [ClF_4_]^−^ and also dissolves the starting material [NEt_3_Me]Cl. Combination of two dichlorofluoromethane solutions containing [NEt_3_Me][ClF_4_] and MeCN⋅BF_3_ leads to the formation of CH_3_CF_3_ (Table 2 Entry 2, for further details see Supporting Information). This is reminiscent of the reaction between succinonitrile and BrF_3_.^27^ We also examined the reactivity towards the tetracyanidoborate anion [B(CN)_4_]^−^. With addition of the Lewis acid BF_3_ we observed the conversion of the cyanido ligands to trifluoromethyl ligands. This result is analogous to the synthesis of [B(CF_3_)_4_]^−^ from [B(CN)_4_]^−^ and ClF_3_ in anhydrous HF.^28^ However, we did not achieve full conversion but a distribution of different borate anions with trifluormethyl and cyanido ligands [B(CF_3_)_*x*_(CN)_4−*x*_]^−^ (Table 2 Entry 3, for further details see Supporting Information). Additionally, we exposed the [BF_3_(CN)]^−^ anion to [NEt_3_Me][ClF_4_] but only fluorination to [BF_4_]^−^ was observed. The reaction of [NEt_3_Me][ClF_4_] with dicyanidoaurate(I) [Au(CN)_2_]^−^ is not selective, however, we were able to identify one Au‐CF_3_ containing product in the reaction mixture via NMR spectroscopy, that is, *cis*‐[AuF_2_(CF_3_)(CN)]^−^ (Table 2 Entry 4, for further details see Supporting Information). Another promising application for [NEt_3_Me][ClF_4_] is the dissolution of noble metals such as gold. After the addition of a piece of elemental gold, the solution containing [ClF_4_]^−^ turns yellow. The ^19^F NMR spectroscopic analysis reveals the formation of mainly [AuF_4_]^−^ (Table 2 Entry 5, for further details see Supporting Information) and traces of other chloridofluoridoaurates ([AuF_3_Cl]^−^ and *cis*‐[AuF_2_Cl_2_]^−^ see Figure S12). Exposure of [NEt_3_Me][ClF_4_] in propionitrile to an atmosphere of CO results in the formation of carbonyl fluoride (COF_2_) within 30 min (Table 2 Entry 6, for further details see Supporting Information).

In conclusion, we developed a facile and fast synthetic procedure to obtain a soluble source of highly reactive [ClF_4_]^−^ in the form of [NEt_3_Me][ClF_4_] avoiding gaseous ClF_3_ which tends to react explosively when exposed to organic matter. We characterized this compound by NMR and Raman spectroscopy and, additionally, single crystal X‐Ray diffraction for the analogous [NEt_4_][ClF_4_]. Furthermore, we presented the first structural and ^19^F NMR spectroscopic proof of the [ClF_2_]^−^ anion. All experimental results are supported by quantum‐chemical calculations. Additionally, we showed several applications of [NEt_3_Me][ClF_4_] as a highly reactive fluorinating agent for the transformation of aryl disulfides into the corresponding pentafluorosulfanyl aryls, nitriles and cyanido complexes into the corresponding trifluoromethyl compounds, carbon monoxide into carbonyl fluoride and the dissolution of elemental gold. In further studies we will explore a broader substrate scope to develop a widely applicable fluorinating reagent for organic and inorganic chemists.

**Caution**! Fluorine, even under dilute conditions, is extraordinarily reactive and can react violently with organic materials under the formation of HF. Similarly, tetrafluoridochlorate(III) and difluoridochlorate(I) are strongly oxidizing compounds, which can decompose violently under certain conditions when exposed to organic materials. Exposure to acidic compounds (e.g. water or boron trifluoride) greatly enhances the reactivity due to the in situ formation of ClF_3_. Additionally, precipitation also greatly enhances the reactivity of tetrafluoridochlorate(III) and difluoridochlorate(I) compounds, leading to explosions at temperatures above −40 °C. Usage of PFA, FEP or PTFE may lower the risk of injury.

## Conflict of interest

The authors declare no conflict of interest.

## Supporting information

As a service to our authors and readers, this journal provides supporting information supplied by the authors. Such materials are peer reviewed and may be re‐organized for online delivery, but are not copy‐edited or typeset. Technical support issues arising from supporting information (other than missing files) should be addressed to the authors.

Supplementary
