# Supplementary material for: Improved Access to Organo‐Soluble Di‐ and Tetrafluoridochlorate(I)/(III) Salts
Source: Angew Chem Int Ed Engl. 2020 Jul 15;59(37):16002–6. doi: 10.1002/anie.202006268 (PMC7540313; doi:10.1002/anie.202006268)
Supplement: Supplementary file 1 — Supplementary [file ANIE-59-16002-s001.pdf]

Angewandte Chemie
Eine Zeitschrift der Gesellschaft Deutscher Chemiker

Supporting Information

Improved Access to Organo-Soluble Di- and Tetrafluoridochlorate-(I)/(III) Salts

*P. Pröhm, J. R. Schmid, K. Sonnenberg, P. Voßnacker, S. Steinhauer, C. J. Schattenberg, R. Müller, M. Kaupp, and S. Riedel**

anie_202006268_sm_miscellaneous_information.pdf

Supporting Information

Table of Contents

Experimental Section 3

Reactivity Studies 7

Crystallographic Section 15

Computational Section 16

References 17

Caution!

Fluorine, even under dilute conditions, is extraordinarily reactive and can react violently with organic materials under the formation of HF. Similarly, tetrafluoridochlorate(III) and difluoridochlorate(I) are strongly oxidizing compounds, which can decompose violently under certain conditions when exposed to organic materials. Exposure to acidic compounds (e.g. water or boron trifluoride) greatly enhances the reactivity due to the *in-situ* formation of ClF_3. Additionally, precipitation also greatly enhances the reactivity of tetrafluoridochlorate(III) and difluoridochlorate(I) compounds, leading to explosions at temperatures above −40 °C. Usage of PFA, FEP or PTFE may lower the risk of injury.

General Information

All experiments were performed under rigorous exclusion of moisture and oxygen using standard Schlenk techniques. Solids were handled in a dry box under argon atmosphere (O_2 < 0.5 ppm, H_2O < 0.5 ppm). HF addition experiments were performend in 3.8 mm PFA tubes with a stainless steel vacuum line. Acetonitrile and propionitrile were dried over Sicapent® prior to use. $[NEt_4]Cl$ and $[NEt_3Me]Cl$ were dried over night at 120 °C under dynamic vacuum. All other chemicals were used as purchased. ClF was synthesized according to literature.[1]
Raman spectra were recorded on a Bruker MultiRAM II equipped with a low-temperature Ge detector (1064 nm, 30-80 mW, resolution, 4 cm^{-1}). NMR spectra were recorded on a JEOL 400 MHz ECS or ECZ spectrometer. All reported chemical shifts are referenced to the Ξ values given in IUPAC recommendations of 2008 using the ^2H signal of the deuterated solvent as internal reference.[2] For external locking acetone-d6 was flame sealed in a glass capillary and the lock oscillator frequency was adjusted to give $\delta(^1H)$ = 7.26 ppm for a $CHCl_3$ sample locked on the capillary. Crystal data were collected on a Bruker D8 Venture diffractometer with a Photon 100 CMOS area detector with MoK_α radiation. Single crystal were picked at –80 °C under nitrogen atmosphere and mounted on a 0.15 mm Mitegen micromount using perfluoroether oil diluted with perfluorohexane. The structures were solved with the ShelXT[3] structure solution program using intrinsic phasing and refined with the ShelXL[4] refinement package using least squares minimizations by using OLEX2.[5] For visualization the Diamond V3.0 program was used.[6]
Structure optimizations and nonrelativistic shielding calculations for the $[XF_n]^-$ (X = Cl, Br, I; n=2, 4, 6) anions and the $CFCl_3$ reference standard were performed using a developers' version of the TURBOMOLE program, release 7.4.[7] Overall four sets of structures were optimized using def2-TZVPPD[8] basis sets (including an effective core potential, ECP, for iodine[8]) and TURBOMOLE standard grid setting 3. Two sets of structures were obtained at the BP86[9]-D3(BJ)[10] level, either in the gas phase or using the Conductor-like Screening Model (COSMO)[11] with r_{solv} = 2.76 [Å] and ε = 35.94 (parameters for the solvent radius and finite permittivity of acetonitrile).

The other two sets were obtained at the B3LYP[12]-D3(BJ)[10] level of theory, also without and with COSMO solvent model. D3(BJ) stands for D3 atom-pairwise dispersion corrections with Becke-Johnson damping.[10]

Subsequent nonrelativistic nuclear-shielding calculations at these structures used a recent improved DFT-GIAO[13] implementation in Turbomole,[14] extended to allow the use of local hybrid functionals.[15] We used two functionals, which had previously been demonstrated to provide accurate ^{19}F shieldings,[15,16] the global hybrid BHLYP,[12b,17] and the LH12ct-SsifPW92.[18] local hybrid functional with position-dependent exact-exchange admixture.[19] These computations used pcSseg-4 basis sets[20] for F, Cl, and Br and ANO-RCC-unc[21] basis sets for I. For each of the abovementioned sets of structures, shielding calculations with these two functionals were performed without or with COSMO. This allows the evaluation of the role of solvent effects acting either indirectly via the structure or directly. The nonrelativistic shielding/shift results are given in Tables S3, S4.

To evaluate the role of spin-orbit and scalar relativistic effects, we also carried out four-component relativistic computations (at the BP86-D3(BJ)(COSMO,CH_3CN)/def2-TZVPPD optimized structures) using the matrix Dirac-Kohn-Sham (mDKS) method[22] implemented in the ReSpect program, version 5.1.0.[23] As neither local hybrids nor BHLYP are currently available for shielding computations in ReSpect, we used a modified B3LYP functional with 50% admixture of exact Hartree-Fock exchange (B3LYP50), which should give results that are close to BHLYP data. Uncontracted Dyall valence quadruple-ζ (Dyall-VQZ)[24] basis sets were used for all atoms. Relativistic shielding/shift results are shown in Table S5.

Experimental Section

Synthesis of $[NEt_3Me][ClF_4]$ in solution (low concentration)

$[NEt_3Me]Cl$ (100 mg, 0.659 mmol, 1 eq.) was dissolved in dried acetonitrile or propionitrile or $CHFCl_2$ (1 ml) and cooled to –35 °C (MeCN) or –55 °C (EtCN, $CHFCl_2$). Dilute F_2 (10 % in Ar) was bubbled through the solution (16 min, 20 ml·min^{-1}, 2 eq). Pure Ar (20 ml·min^{-1}) was bubbled through the solution for 15 min in order to remove residual amounts of reactive gases. A colorless solution was obtained.
^{1}H NMR (400 MHz, EtCN, ext. acetone-d6, 20 °C) δ[ppm]= 3.87 (q, $^3J(^1H,^1H)$=7.30 Hz, 6H, CH_2) 3.48 (s, 3H N-CH_3), 1.85 (t, $^3J(^1H,^1H)$=7.30 Hz, $^3J(^{14}N,^1H)$=1.84 Hz, CH_3). ^{19}F NMR (377 MHz, EtCN, ext. acetone-d6, 20 °C) δ[ppm]= 67 (ClF_4^-); FT-Raman (EtCN, −196 °C): $\tilde{\nu}$= $[ClF_4]^-$: 500 (a_{1g}), 407(b_{1g}), 278 cm^{-1} (b_{2g}).

Synthesis of $[NEt_3Me][ClF_4]$ in solution (high concentration)

$[NEt_3Me][Cl_3]$ was prepared by the addition of 1 eq. of Cl_2 to $[NEt_3Me]Cl$. $[NEt_3Me][Cl_3]$ (0.621 g, 2.79 mmol, 1 eq.) was diluted with acetonitrile (0.3 ml) and cooled to –30 °C. Dilute F_2 (10 % in Ar) was bubbled through the solution (68 min, 20 ml min^{-1}, 2 eq). To remove residual amounts of reactive gases, Ar was bubbled through the solution for (15 min, 20 ml min^{-1}) . A colorless solution was obtained. Analytics was identical to samples with low concentration.

Crystal growth of $[NEt_4][ClF_4]$

The synthetic procedure is similar to $[NEt_3Me][ClF_4]$. Single crystals were obtained by slowly cooling a propionitrile solution to –80 °C over 1 week in a freezer. An ethanol bath in a Dewar was used to reduce the cooling rate.

Synthesis of $[NEt_4]_3[ClF_4][ClF_2]_2$ in solution

$[NEt_4]Cl$ (300 mg, 1.81 mmol, 1 eq.) was dissolved in acetonitrile (3 ml) and cooled to –30 °C. Dilute F_2 (10 % in Ar) was bubbled through the solution (20 min, 20 ml min^{-1}, 1.2 eq). To remove residual amounts of reactive gases, Ar was bubbled through the solution for 15 min. A colorless solution was obtained.
^{1}H NMR (400 MHz, MeCN, ext. acetone-d6, −40 °C) δ[ppm]= 3.11 (q, $^3J(^1H,^1H)$=7.18 Hz, 8H, CH_2), 1.12 (t, $^3J(^1H,^1H)$=7.18 Hz, $^3J(^{14}N,^1H)$=1.84 Hz, 12H, CH_3). ^{19}F NMR (377 MHz, MeCN, ext. acetone-d6, −40 °C) δ[ppm]= 67 (s, $[ClF_4]^-$), −125 (s, $[ClF_2^-]$) FT-Raman (MeCN, −196 °C): $\tilde{\nu}$= $[ClF_4]^-$: 500 (a_{1g}), 407(b_{1g}), 278 (b_{2g}), $[ClF_2]^-$: 455 cm^{-1} (a_{1g}).

Crystal growth of $[NEt_3Me]_3[ClF_4][ClF_2]_2$

The synthetic procedure is similar to $[NEt_4]_3[ClF_4][ClF_2]_2$. Single crystals were obtained by slowly cooling a propionitrile solution to –35 °C over 2 days in a freezer. An ethanol bath in a Dewar was used to reduce the cooling rate.

Synthesis of [NEt_3Me][ClF_2] in solution

[Net3Me]Cl (50 mg, 0,330 mmol, 1 eq) was dissolved in propionitrile (0.5 ml) and cooled to −50 °C. Dilute ClF (10 % in Ar) was bubbled through the solution (8.5 min, 20 ml min^{-1}, 2 eq). To remove residual amounts of reactive gases, Ar was bubbled through the solution for 15 min. A colorless solution was obtained. The reaction mixture was allowd to warm to −10 °C and subsequently slowly cooled to −80 °C in an ethanol bath Dewar to reduce the cooling rate. Single crystals were obtained after 4 days.

Raman (crystal, −196 °C): $\tilde{\nu}$= [ClF_2]$^-$: 457 cm^{-1} (a_{1g}).

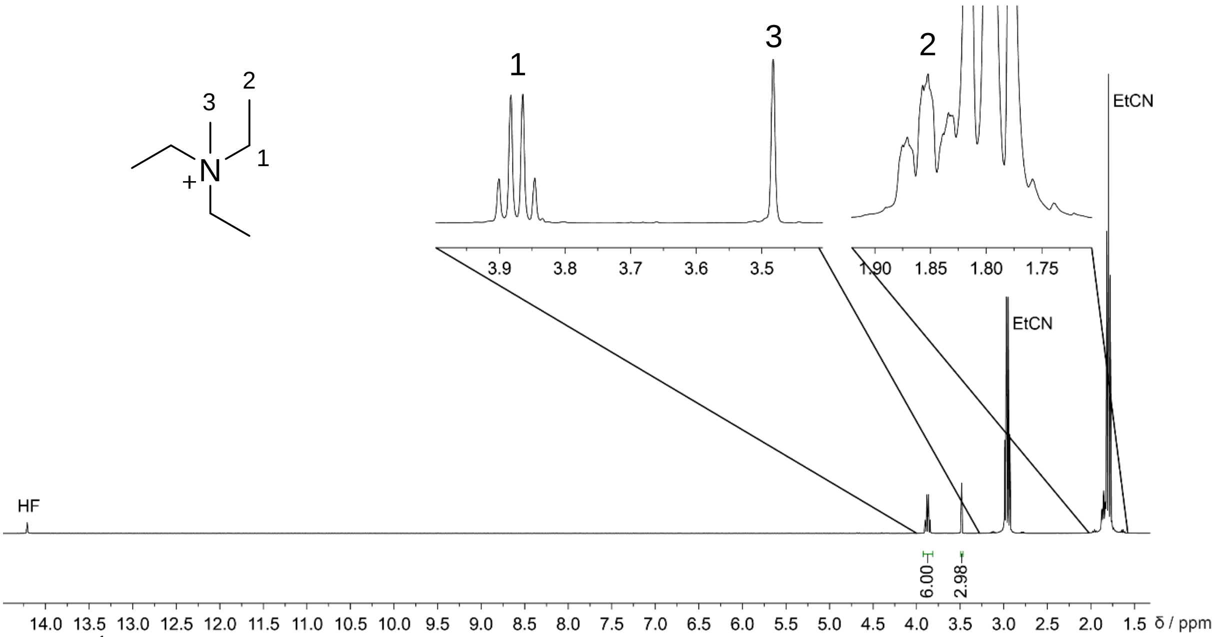

Figure S1. ^{1}H NMR spectrum (400 MHz, EtCN, ext. acetone-d6, 21 °C) of a solution of [NEt_3Me][ClF_4] in propionitrile.

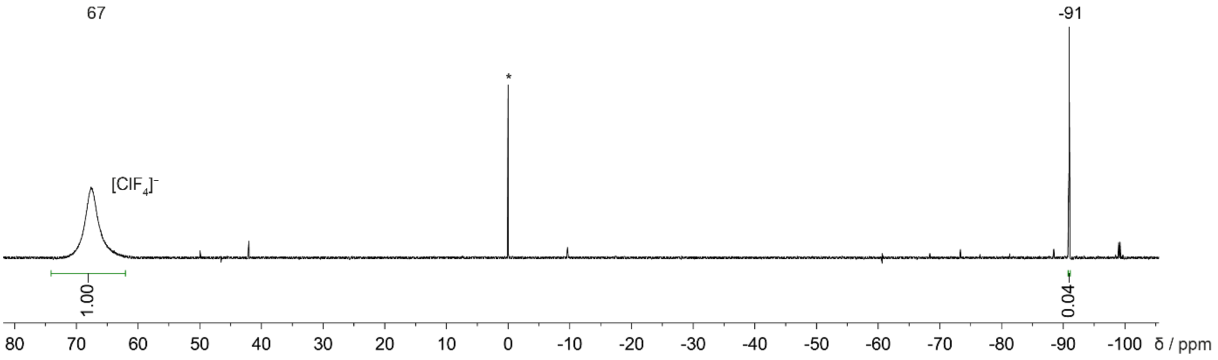

Figure S2. ^{19}F NMR spectrum (377 MHz, EtCN, ext. acetone-d6, 21 °C) of a solution of [NEt_3Me][ClF_4] in acetonitrile. Asterisk (*) denotes $CFCl_3$. Unknown impurity at −91 ppm (t).

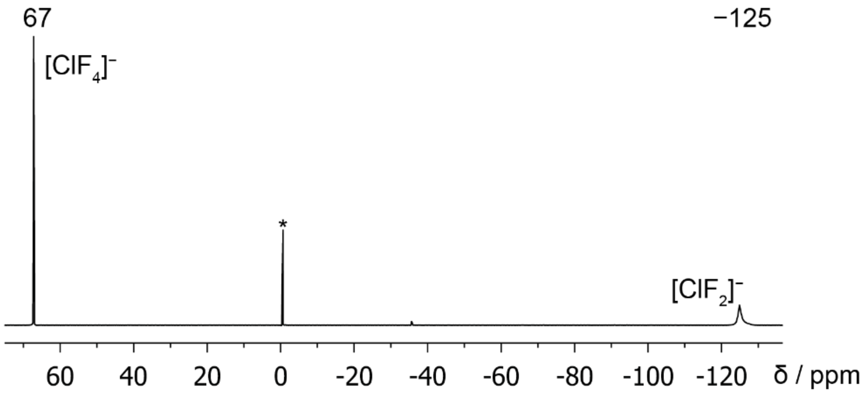

Figure S3. ^{19}F NMR spectrum (377 MHz, MeCN, ext. acetone-d6, −40 °C) of a solution of $[NEt_3Me]_3[ClF_4][ClF_2]_2$ in acetonitrile. Asterisk (*) denotes $CFCl_3$.

We realized that the ^{19}F NMR signal of $[NEt_3Me][ClF_4]$ shows different line broadening under certain conditions. Therefore, we studied the influence of temperature and HF impurities on the line width. Figure S4 shows the ^{19}F NMR spectra at –20 °C and –40 °C prior and after addition of 1 eq of HF. Raising the temperature from –40 °C to –20 °C leads to a significant line broadening (–40 °C : FWHM = 164.8 Hz, –20 °C = 414.8 Hz). Addition of HF also leads to line broadening at –20 °C and –40 °C (–40 °C + HF addition: FWHM = 251.6 Hz; –20 °C + HF addition: FWHM = 637.7 Hz).

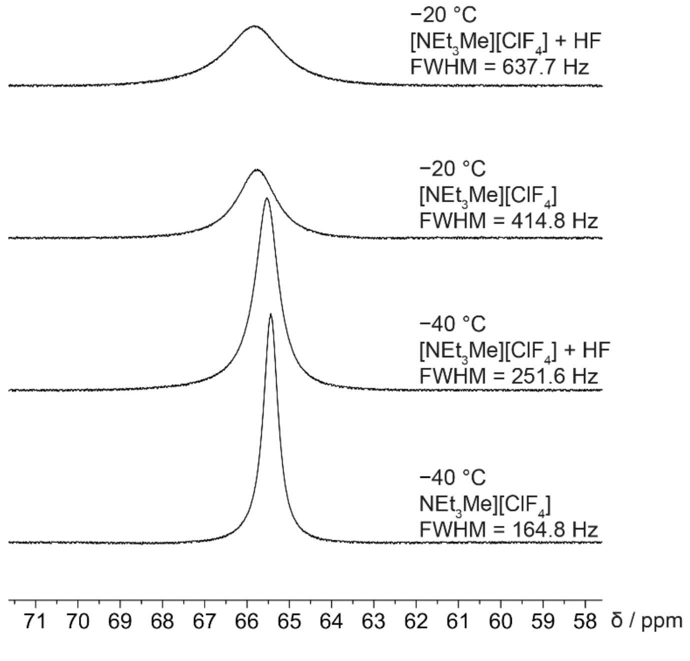

Figure S4. ^{19}F NMR spectra (377 MHz, EtCN, ext. acetone-d6) of a solution of $[NEt_3Me][ClF_4]$ in propionitrile at –20 °C and –40 °C prior and after addition of 1 eq HF.

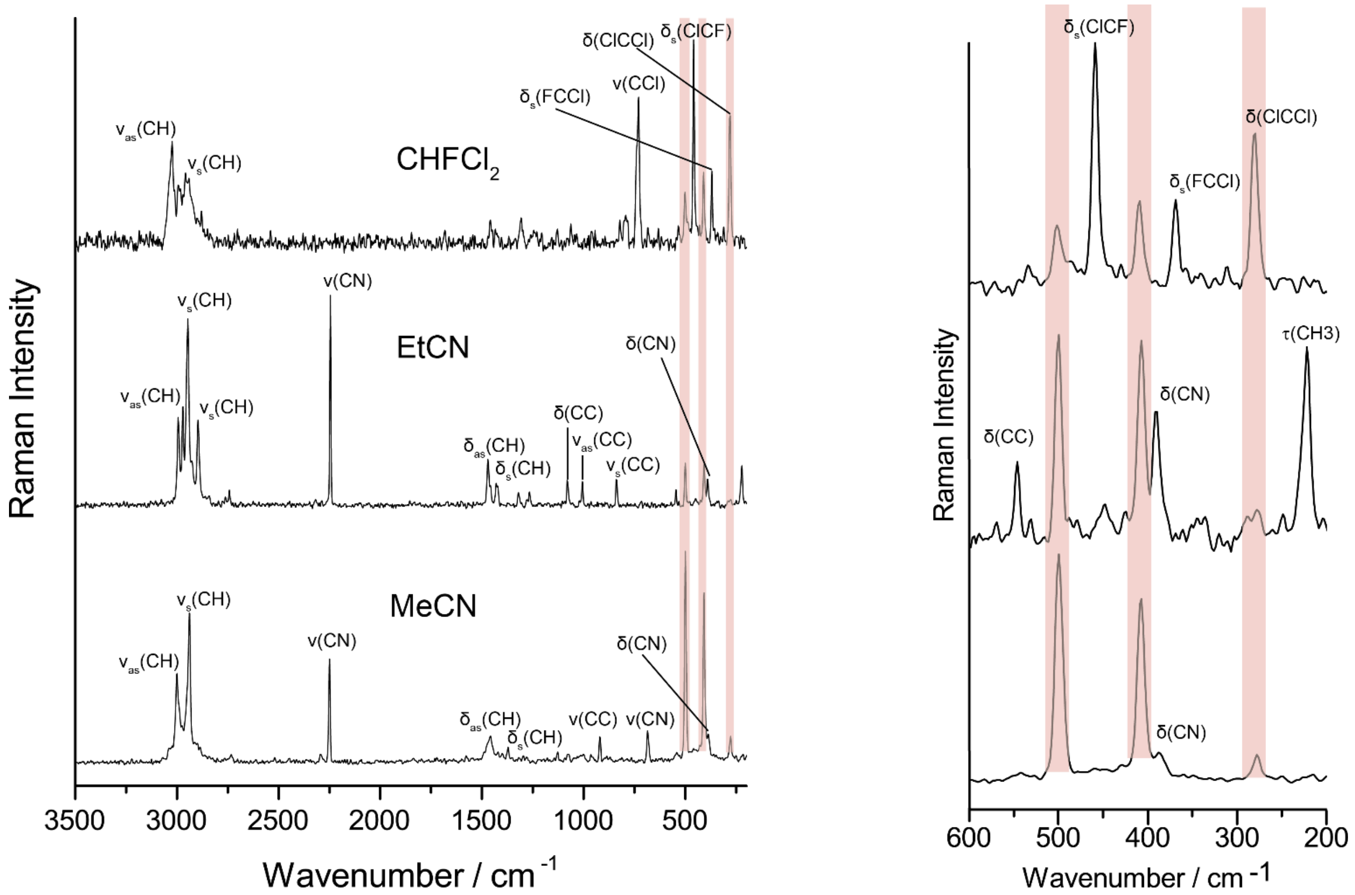

Figure S5. Raman spectra (–196 °C) of [NEt_3Me][ClF_4] in different solvents: $CHFCl_2$ (top), EtCN (middle), MeCN (bottom). Left: full spectrum, right: Extract from 200-600 cm^{-1}. [ClF_4]$^-$ bands are highlighted in red.

[NEt_3Me][ClF_4] in different solvents, $CHFCl_2$ (top), propionitrile (middle) and acetonitrile (bottom). The three characteristic bands for [ClF_4]$^-$ (500 cm^{-1} (a_{1g}), 407 cm^{-1} (b_{1g}), 278 cm^{-1} (b_{2g})) are highlighted in red. The different intensities are due to different concentrations. In case of propionitrile the concentration is too low (0.32 mol·l^{-1}) to observe the b_{2g} vibration. In case of $CHFCl_2$ the band of the b_{2g} vibration is superimposed by a solvent band.

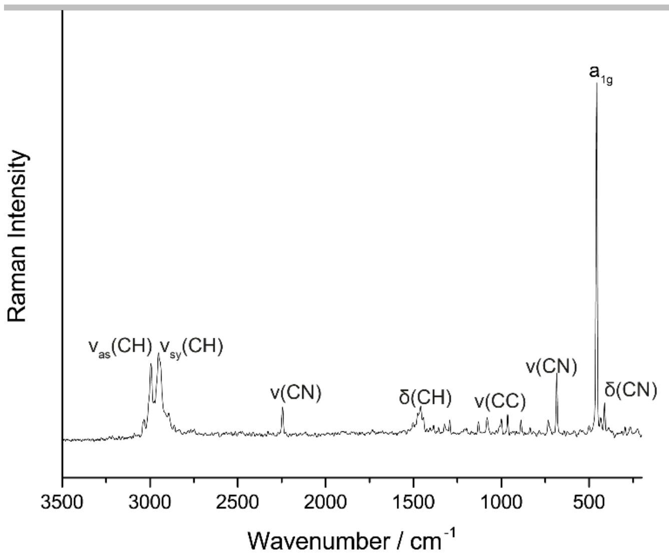

Figure S6. Raman spectrum (–196 °C) of $[NEt_3Me][ClF_2]$. Anion band a_{1g} 457 cm^{-1}.

Reactivity Studies

Disulfide activation

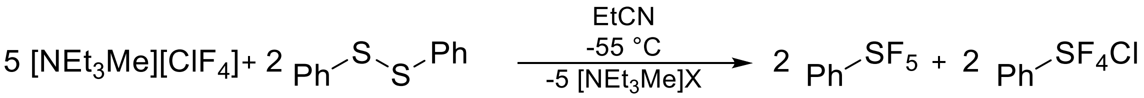

To a cooled (−55 °C) solution of $[NEt_3Me][ClF_4]$ in propionitrile (1 ml, 0.659 mol·l^{-1}, 1 eq.) a solution of phenyl disulphide (28.8 mg, 0.132 mmol, 0.2 eq) in propionitrile (2 ml) was added dropwise. The solution was stirred for 15 min at −55 °C, then allowed to warm to r.t. and stirred over night. A 1 : 4 : 2 mixture of Ph-SF_5 : *cis*-$PhSF_4Cl$: *trans*-$PhSF_4Cl$ was obtained. After addition of H_2O (0.5 ml) *trans*-$PhSF_4Cl$ was selectively hydrolysed to $PhSO_2F$. Addition of dilute potassium hydroxide solution (1 ml) and heating to 90 °C over night led to the hydrolysis of *cis*-$PhSF_4Cl$ and $PhSO_2F$. Products were identified by ^{19}F NMR spectroscopy. NMR yield $PhSF_5$: 17 %
^{19}F NMR (377 MHz, EtCN, ext. acetone-d6, 21 °C) δ= 85 (quin. $^{2}J(^{19}F,^{19}F)$=148Hz, 1F, $PhSF_5$ *trans*-F), 63 (d, 4F, $PhSF_5$ *cis*-F), 165 (td, $^{2}J(^{19}F,^{19}F)$=164Hz, $^{2}J(^{19}F,^{19}F)$=149 Hz, 1F, *cis*-$PhSF_4$ *trans*-F), 100 (dd, $^{2}J(^{19}F,^{19}F)$=81 Hz, 2F, *cis*-$PhSF_4$ *cis*-F), 67 (dt, 2F, *cis*-$PhSF_4$ *cis*-F), 137 (s, 4F, *trans*-$PhSF_4Cl$), 64 ppm (s, 1F $PhSO_2F$).

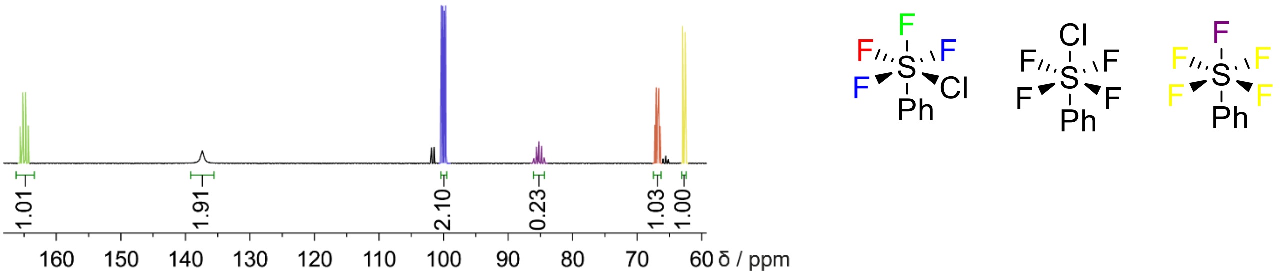

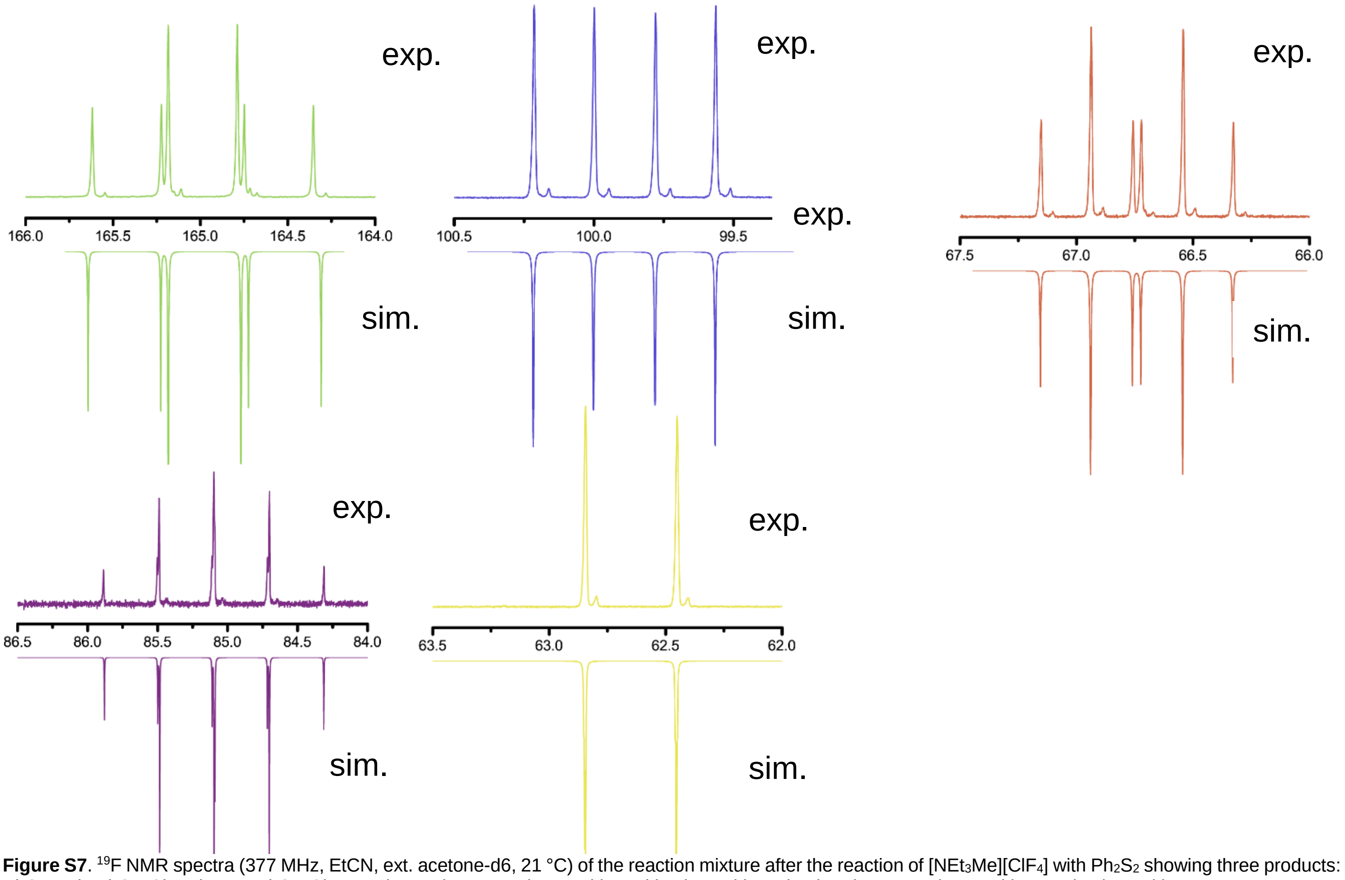

Figure S7. ^{19}F NMR spectra (377 MHz, EtCN, ext. acetone-d6, 21 °C) of the reaction mixture after the reaction of [NEt_3Me][ClF_4] with Ph_2S_2 showing three products: $PhSF_5$, *cis*-$PhSF_4Cl$ and *trans*-$PhSF_4Cl$. Experimental spectra shown with positive intensities, simulated spectra shown with negative intensities.

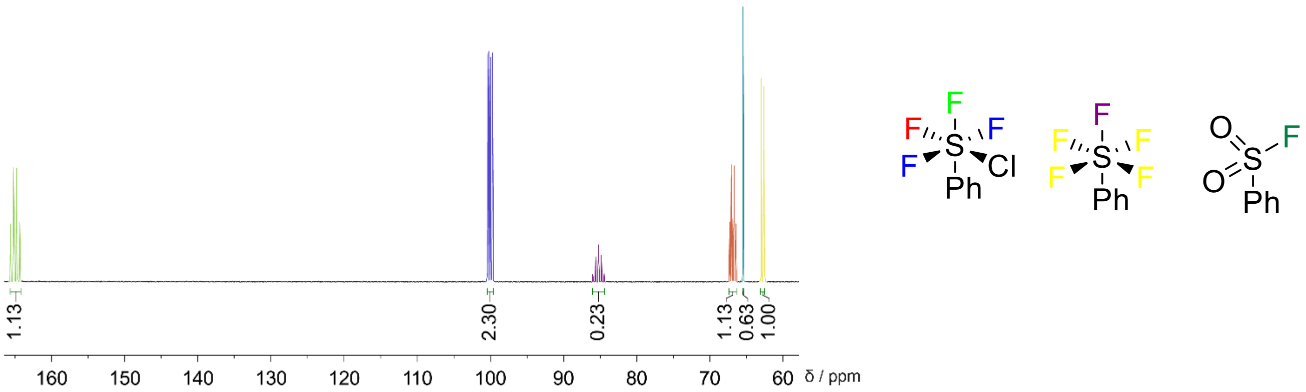

Figure S8. ^{19}F NMR spectrum (377 MHz, EtCN, ext. acetone-d6, 21 °C) of the hydrolysed reaction mixture (H_2O, r.t., 15 min) after the reaction of [NEt_3Me][ClF_4] with Ph_2S_2 showing three products: $PhSF_5$, *cis*-$PhSF_4Cl$ and $PhSO_2F$.

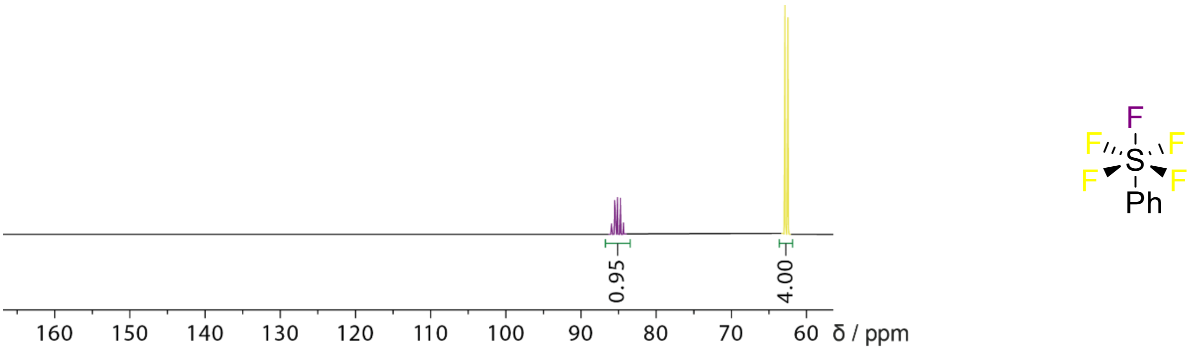

Figure S9. ^{19}F NMR spectrum (377 MHz, EtCN, ext. acetone-d6, 21 °C) of the hydrolysed reaction mixture (KOH_{aq}, 90 °C, 8 h) after the reaction of $[NEt_3Me][ClF_4]$ with Ph_2S_2 showing only $PhSF_5$.

Nitrile activation

$$2\,[NEt_3Me][ClF_4] + 2\,MeCN \cdot BF_3 \xrightarrow[\substack{-2\,[NEt_3Me][BF_4] \\ -N_2 \\ -Cl_2}]{\substack{CHFCl_2 \\ -55\ °C}} 2\,CH_3CF_3$$

The acetonitrile boron trifluoride complex was synthesized by the addition of BF_3 to acetonitrile at r.t. followed by removal of residual acetonitrile under reduced pressure.
To solid acetonitrile boron trifluoride complex (35.9 mg, 0.330 mmol, 0.5 eq) a solution of $[NEt_3Me][ClF_4]$ (150 mg, 0.659 mmol, 1 eq) in dichlorofluoromethane (1 ml) was added at –60 °C. The reaction mixture was analyzed by ^{19}F NMR spectroscopy (Figure S9). The reaction equation suggests the equimolar formation of CH_3CF_3 and $[BF_4]^-$. The ^{19}F NMR spectrum shows a ratio of 1:230 in favour of $[BF_4]^-$this can be rationalised due to several reasons: First, CH_3CF_3 has a boiling point of –50 °C. During the reaction and the transfer of the reaction mixture probably significant amounts of the product evaporated. Secondly, during the reaction highly reactive ClF_3 is formed leading to side products which are evident in the ^{19}F NMR spectrum. The signal at –129 ppm can be identified as $[SiF_6]^{2-}$ from a reaction with the glass vessle.

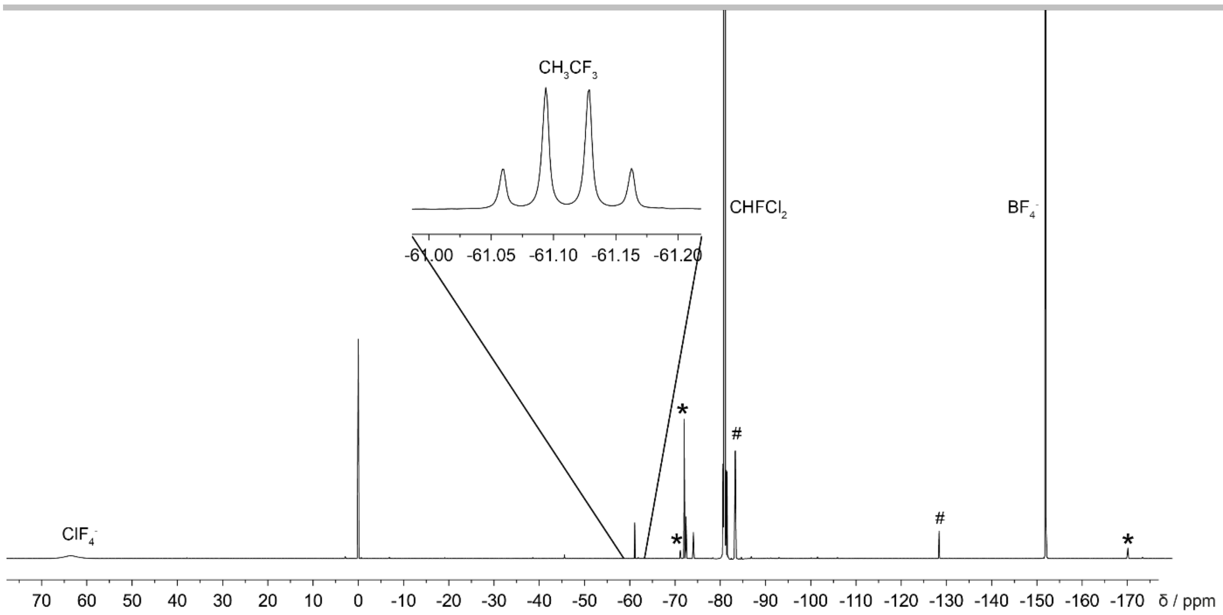

Figure S10. ^{19}F NMR spectrum (377 MHz, EtCN, ext. acetone-d6, 21 °C) of the reaction mixture. Symbols denote solvent impurities (*), side products generated during the reaction (#).

CO activation

$$[NEt_3Me][ClF_4] + CO \xrightarrow[-[NEt_3Me][ClF_2]]{EtCN} COF_2$$

A solution of $[NEt_3Me][ClF_4]$ (150 mg, 0.659 mmol, 1 eq) was stirred under a CO atmosphere for 30 min at r.t. Then the gas-phase was analyzed via FT-IR spectroscopy. The FT-IR spectrum (Figure S7) only shows the presence of starting material (CO) solvent (EtCN) and product (COF_2). The formation of $COCl_2$ or COClF was not observed.

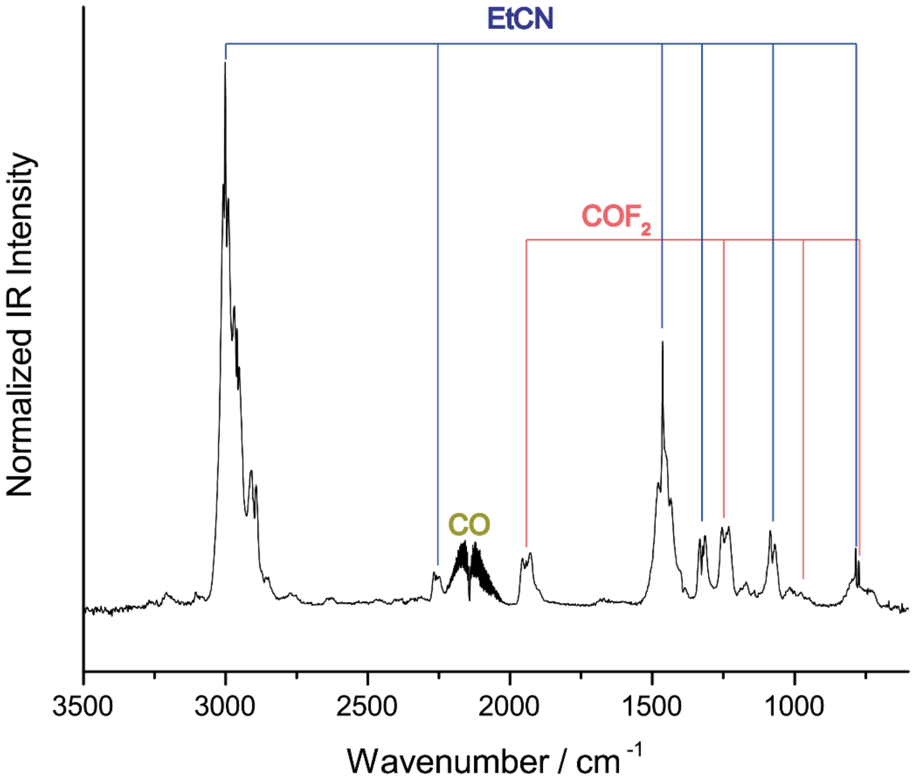

Figure S11. Gas-phase FT-IR spectrum (50 mbar, 10 cm) of the reaction mixture showing only solvent (EtCN, blue), starting material (CO, green) and product (COF_2, red).

Dissolution of gold

$$2\,[NEt_3Me][ClF_4] + 2\,Au \xrightarrow[-Cl_2]{MeCN} 2\,[NEt_3Me][AuF_4]$$

Gold metal (16 mg) was dissolved over 2 days in a solution of $[NEt_3Me][ClF_4]$ in acetonitrile (0.5 ml, 8 mol·l^{-1}) at r.t. The solution was analyzed with ^{19}F NMR spectroscopy. The major product is $[AuF_4]^-$ (95 %) but traces of $[AuF_3Cl]^-$ (3%) and *cis*-$[AuF_2Cl_2]^-$ (2 %) were also present in the reaction mixture.

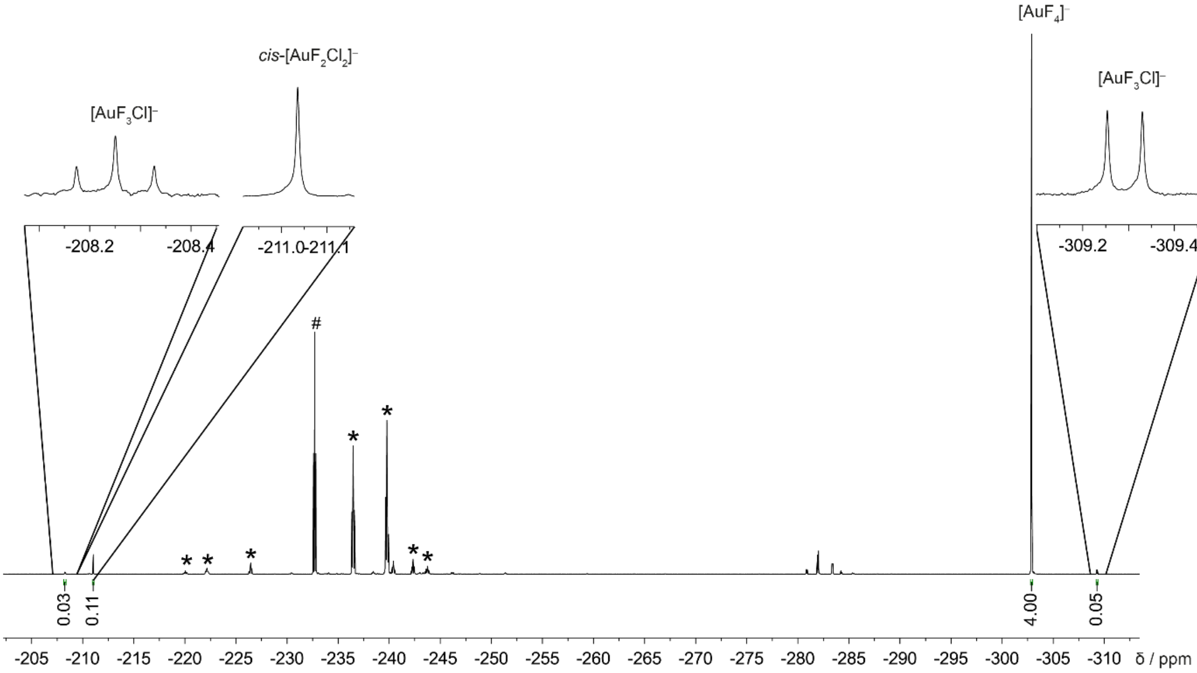

Figure S12. ^{19}F NMR spectrum (377 MHz, MeCN, ext. acetone-d6, 21 °C) of the reaction mixture after the reaction of $[NEt_3Me][ClF_4]$ with Au. Symbols denote fluorinated impurities: # fluorinated solvent (FCH_2CN), * halogenated cations.

Tetracyanoborate activation

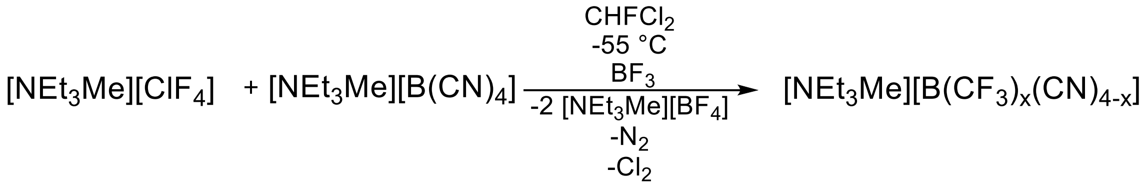

Potassiumtetracyanoborate (500 mg, 3.25 mmol, 1 eq) was dissolved in acetonitrile (10 ml). A solution of triethylmethylaommonium chloride (0.493 mg, 3.25 mmol, 1 eq) in acetonitrile (5 ml) was added. The colourless precipitated was filtrated and the solvent was removed under reduced pressure to yield tetramethylammonium tetracyanoborate.

Triethylmethylammonium tetracyanoborate (76.0 mg, 0.330 mmol, 0.5 eq) was dissolved in $CHFCl_2$ (1 ml) and boron trifluoride (67,0 mg, 0.989 mmol, 1.5 eq) was added. The solution was cooled to −60 °C. A cooled solution of Triethylmehtylammonium tetrafluoridochlorate(III) (1 ml, 0.659 mol*l^{-1}, 1 eq) was added at −60 °C, stirred for 5 min at −60 °C and let allowd to warm to r.t. Volatiles were removed and the residue was dissolved in CD_3CN and analyzed via ^{19}F and ^{11}B NMR spectroscopy. $[B(CF_3)_x(CN)_{4-x}]^-$ anions were identified according to literature.

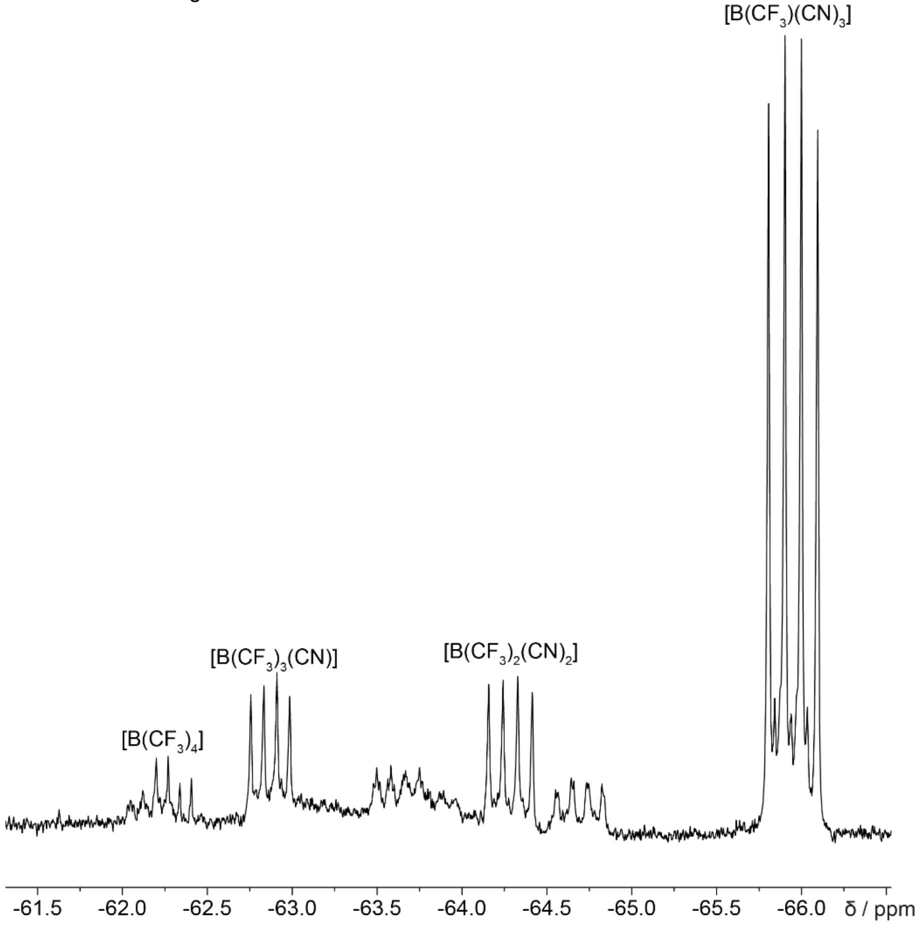

Figure S13. ^{19}F NMR spectrum (377 MHz, CD_3CN, 21 °C) of the reaction mixture after the reaction of $[NEt_3Me][ClF_4]$ with $[NEt_3Me][B(CN)_4]$.

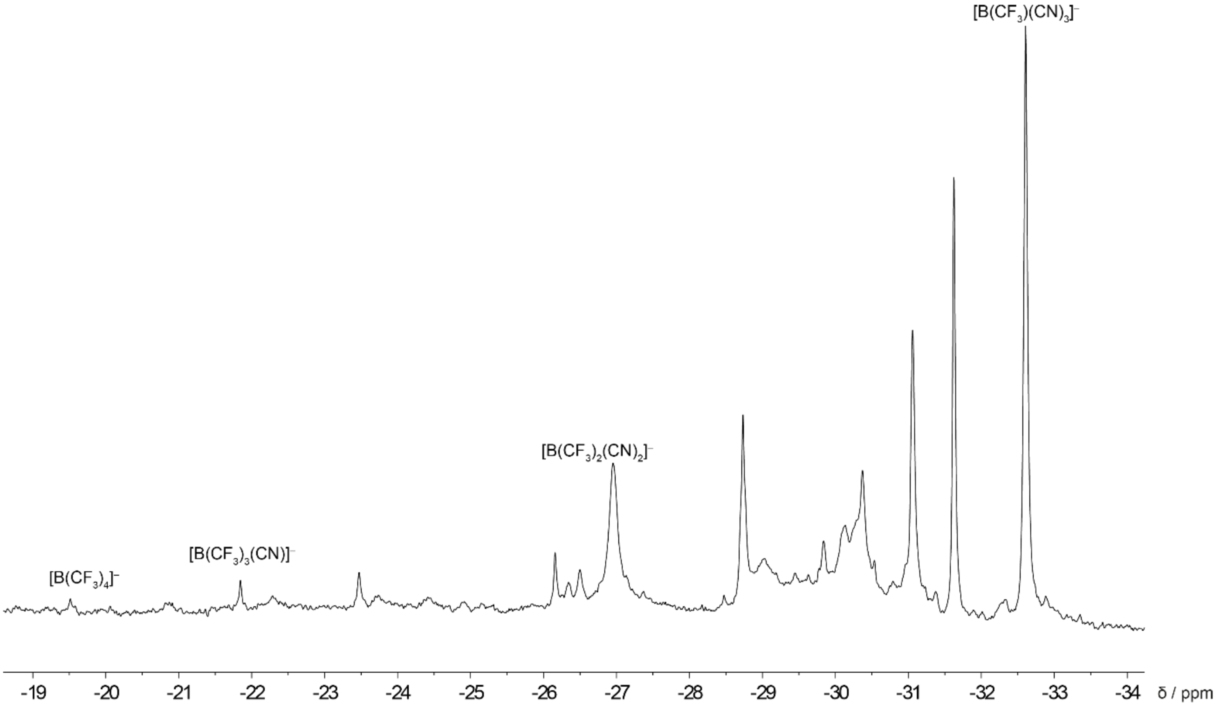

Figure S14. $^{11}B\{^{19}F\}$ NMR spectrum (129 MHz, CD_3CN, 21 °C) of the reaction mixture after the reaction of $[NEt_3Me][ClF_4]$ with $[NEt_3Me][B(CN)_4]$.

Difluoridoaurate activation

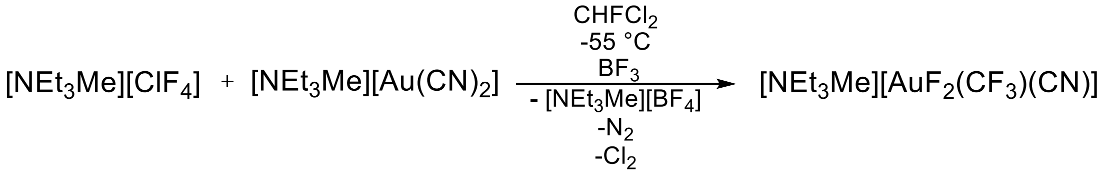

Cation metathesis was achived in a simiar procedure to triethylmethylammonium tetracyanoborate.
Triethylmethylammonium dicyanoaurate (43 mg, 0.118 mmol, 0.18 eq) was dissolved in $CHFCl_2$ (1 ml). Boron trifluoride (19 mg, 0.280 mmol, 0.42 eq) was added. The solution was cooled to −60 °C. A cooled solution of triethylmehtylammonium tetrafluoridochlorate(III) (1 ml, 0.659 mol*l^{-1}, 1 eq) was added at −60 °C, stirred for 5 min at −60 °C and let allowd to warm to r.t and analyzed via ^{19}F NMR spectroscopy.
Cis-cyanodifluorido(trifluoromethyl)aurate(III) was identfied using ^{19}F NMR spectroscopy in comparison with literature values.[25] Due to the low concentration of *cis*-cyanodifluorido(trifluoromethyl)aurate(III) the region between −80 and −170 ppm in the NMR spectrum is not shown. It contains solvent signal $CHFCl_2$ at −81 ppm, $[SiF_6]^{2-}$ at −129 ppm and $[BF_4]^-$ at −152 ppm
δ[ppm]= -58.29 (dd, $^3J(^{19}F, ^{19}F)$ = 5.8 Hz, $^3J(^{19}F, ^{19}F)$ = 2.5 Hz, 3F), −196.66 (dq, $^2J(^{19}F, ^{19}F)$ = 65.3 Hz, $^3J(^{19}F, ^{19}F)$ = 2.5 Hz, 1F), −254.31 (dq, $^2J(^{19}F, ^{19}F)$ = 65.3 Hz, $^3J(^{19}F, ^{19}F)$ = 5.8, 1F)

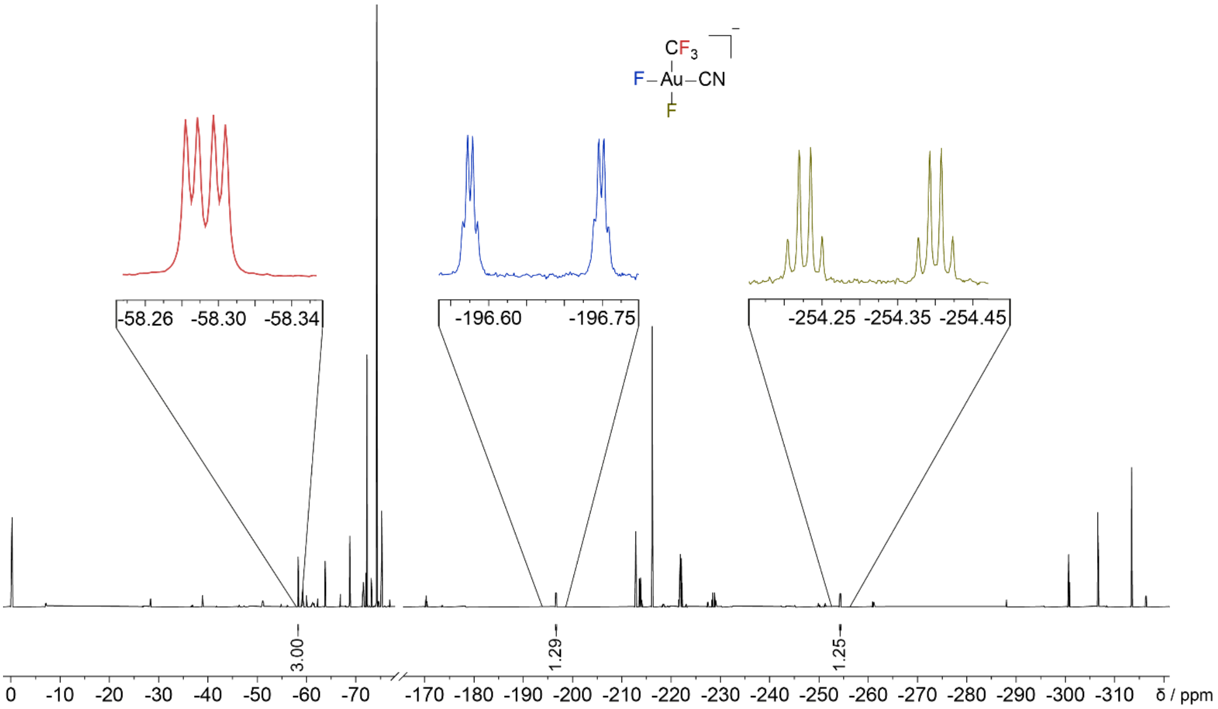

Figure S15. ^{19}F NMR spectrum (377 MHz, $CHFCl_2$, ext. acetone-d6, 21 °C) of the reaction mixture after the reaction of $[NEt_3Me][ClF_4]$ with $[NEt_3Me][Au(CN)_2]$. Omitted spectral area between −80 and −170 ppm contains solvent $CHFCl_2$ (−81 ppm), $[SiF_6]^{2-}$ (−129 ppm) and $[BF_4]^-$ (−152 ppm).

Crystallographic section

Table S1. Crystallographic details of $[NEt_3Me]_3[ClF_4][ClF]_2$ and $[NEt_4][ClF_4]$.

Compound	$[NEt_3Me]_3[ClF_4][ClF]_2$	$[NEt_4][ClF_4]$	$[Net_3Me][ClF_2]$
**Empirical formula**	$C_{21}H_{54}Cl_3F_8N_3$	$C_8H_{20}ClF_4N$	$C_7H_{18}ClF_2N$
**Formula weight**	607.02	241.70	189.67
**Temperature/K**	100.0	99.92	100.0
**Crystal system**	triclinic	monoclinic	monoclinic
**Space group**	$P\bar{1}$	*C*2/*c*	*P*2₁/*c*
***a*/Å**	6.9802(8)	11.4818(9)	11.2608(4)
***b*/Å**	14.6749(16)	7.5474(5)	6.9954(2)
***c*/Å**	15.3580(16)	14.3613(11)	12.6412(4)
***α*/°**	97.190(4)	90	90
***β*/°**	92.017(4)	110.877(3)	102.6650(10)
***γ*/°**	102.608(4)	90	90
**Volume/Å^3**	1520.0(3)	1162.81(15)	971.57(5)
**Z**	2	4	4
**ρ_{calc}g/cm^3**	1.326	1.381	1.297
**μ/mm^{-1}**	0.366	0.347	0.367
**F(000)**	648.0	512.0	408.0
**Crystal size/mm^3**	0.34 × 0.23 × 0.18	0.32 × 0.16 × 0.04	0.293 × 0.216 × 0.154
**Radiation**	MoK$_\alpha$ (λ = 0.71073)	MoK$_\alpha$ (λ = 0.71073)	MoK$_\alpha$ (λ = 0.71073)
**2Θ range for data collection/°**	5.358 to 57.15	6.072 to 56.738	3.708 to 61.112
**Index ranges**	−9 ≤ h ≤ 9, −19 ≤ k ≤ 19, −20 ≤ l ≤ 20	−15 ≤ h ≤ 15, −9 ≤ k ≤ 10, −19 ≤ l ≤ 19	16 ≤ h ≤ 16, -9 ≤ k ≤ 10, -18 ≤ l ≤ 18
**Reflections collected**	85154	25147	24557
**Independent reflections**	7678 [R_{int} = 0.0626, R_{sigma} = 0.0322]	1453 [R_{int} = 0.0452, R_{sigma} = 0.0173]	2961 [R_{int} = 0.0275, R_{sigma} = 0.0157]
**Data/restraints/parameters**	7678/0/331	1453/0/68	2961/0/117
**Goodness-of-fit on F^2**	1.059	1.172	1.065
**Final R indexes [I>=2σ (I)]**	R_1 = 0.0372, wR_2 = 0.0810	R_1 = 0.0300, wR_2 = 0.0752	R_1 = 0.0268, wR_2 = 0.0751
**Final R indexes [all data]**	R_1 = 0.0517, wR2 = 0.0878	R_1 = 0.0334, wR_2 = 0.0766	R_1 = 0.0316, wR_2 = 0.0804
**Largest diff. peak/hole / e Å^{-3}**	0.34/−0.44	0.32/−0.38	0.34/-0.34
**CCDC deposition number**	1948997	1948998	2004243

Table S2. Bond lengths and angles of $[ClF_4]^-$ anions in $[NEt_4][ClF_4]$ and $[NEt_3Me]_3[ClF_4][ClF_2]_2$ in comparison with the literature known compounds $[pyp][ClF_4]$, $[NO][ClF_4]$, $Cs[ClF_4]$, $Rb[ClF_4]$ and $K[ClF_4]$.[26] Lengths in pm, angles in °.

Bond/Angle	$[NEt_4][ClF_4]$	$[NEt_3Me]_3[ClF_4][ClF_2]_2$	$[pip][ClF_4]$	$[NO][ClF_4]$	$Cs[ClF_4]$	$Rb[ClF_4]$	$K[ClF_4]$
**Cl1-F1**	179.3(1)	178.8(1)	175.9(2)	187.4(1)	179.4(4)	180.34(9)	179.82(6)
**Cl1-F2**	180.6(1)	179.4(1)	181.4(1)	172.7(1)	179.2(4)	179.30(9)	-
**Cl1-F3**	-	180.0(1)	177.1(1)	-	-	-	-
**Cl1-F4**	-	180.8(1)	181.3(1)	-	-	-	-
**F1-Cl1-F2**	90.01(5)	89.78(5)			89.1(2)	89.33(4)	89.79(4)
**F2-Cl1-F1'**	89.99(5)	-			90.9(2)	90.67(4)	90.21(4)
**F2-Cl1-F3**	-	89.66(5)			-	-	-
**F3-Cl1-F4**	-	90.21(5)			-	-	-
**F4-Cl1-F1**	-	90.35(5)			-	-	-

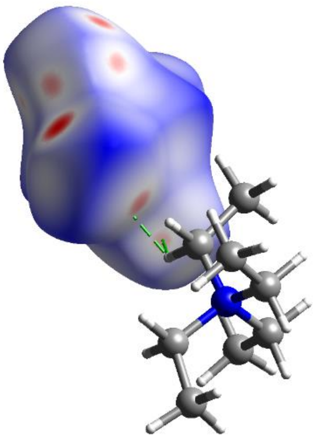

Figure S16. Hirshfeld surface of of the $[ClF_4]^-$ anion in $[NEt_3Me][ClF_4]$. Color code: blue = N, grey = C, white H, green dashed line displays hydrogen bond.[27]

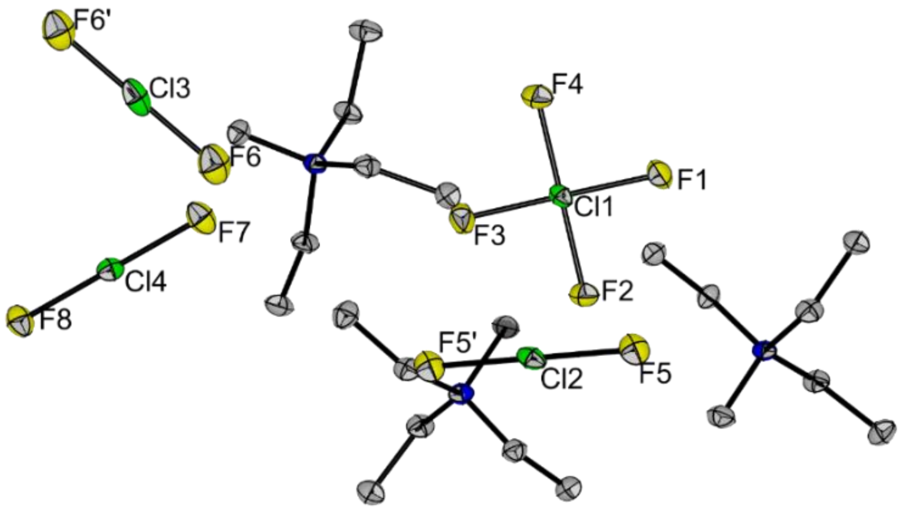

Figure S17. Crystal structure of $[NEt_3Me]_3[ClF_4][ClF_2]_2$. Color code: yellow = F, green = Cl, blue = N, grey = C. Displacement ellipsoids are shown at 50 % probability at 100 K. Selected bond lengths [pm] and bond angles [°]: F1−Cl1 178.8(1), F2−Cl1 179.4(1), F3−Cl1 180.0(1), F4−Cl1 180.8(2), F5−Cl2 185.4(1), F6−Cl3 184.1(2), F7−Cl4 184.9(1), F8−Cl4 185.4(1), F7−Cl4−F8 179.66(5). Hydrogen atoms omitted for clarity.

By slowly cooling a reaction mixture of $[NEt_3Me]Cl$ with 1.2 eq. fluorine in acetonitrile, single crystals of $[NEt_3Me]_3[ClF_4][ClF_2]_2$ were obtained (Figure S16). This compound crystallizes in the space group $P\bar{1}$. In the asymmetric unit are four anionic moieties. One is a $[ClF_4]^-$ anion, the second is an asymmetric $[ClF_2]^-$ (F7Cl4F8) anion. Furthermore, there are two $[ClF_2]^-$ units with the chlorine atom on centers of inversion (F6Cl3F6' and F5Cl2F5') (Figure 4). The bond lengths and angles in the $[ClF_4]^-$ anion in $[NEt_3Me]_3[ClF_4][ClF_2]_2$ are very similar to the ones found in $[NEt_4][ClF_4]$. The Cl−F bond lengths of the $[ClF_2]^-$ anions vary between 184.1(2) pm and 185.4(1) pm. The bond angle of the $[ClF_2]^-$ anion (F7Cl4F7) is 179.66(5) °.

Computational section

Table S3. Calculated ^{19}F NMR chemical shifts relative to $CFCl_3$ (in ppm) of $[XF_n]^-$ (X = Cl, Br, I; n=2, 4, 6) in comparison with experimental values, at BP86-D3 structures.[a]

	BHLYP				LH12ct-SsifPW92				
opt.<br>NMR	gas.<br>gas	gas<br>COSMO	COSMO.<br>gas	COSMO<br>COSMO	gas.<br>gas	gas<br>COSMO	COSMO.<br>gas	COSMO<br>COSMO	δ_{exp}
$[ClF_2]^-$	−193.2	−200.0	−196.6	−202.4	−166.3	−170.1	−171.5	−174.4	-125
$[ClF_4]^-$	80.6	76.1	71.9	67.8	71.8	68.6	63.3	60.5	67
$[ClF_6]^-$	284.3	286.8	275.0	277.6	255.1	257.3	246.6	248.9	–
$[BrF_2]^-$	−284.4	−294.6	−286.2	−295.5	−262.6	−270.7	−265.4	−272.7	−210
$[BrF_4]^-$	−32.2	−38.3	−36.3	−42.0	−36.6	−41.6	−40.7	-45.4	-37
$[BrF_6]^-$	131.9	135.0	126.0	129.2	114.6	117.4	109.0	111.9	94
$[IF_2]^-$	−350.1	−359.3	−351.4	−360.1	−338.1	−346.7	−339.5	−347.8	−282
$[IF_4]^-$	−103.1	−109.5	−105.1	−111.1	−109.8	−115.5	−111.8	−117.3	−106
$[IF_6]^-$	31.1	33.3	28.1	30.5	14.8	16.9	11.9	14.1	13
$\sigma(CFCl_3)$	185.4	189.7	183.8	183.8	195.2	198.8	193.7	197.5	

[a] At BP86-D3(BJ)/def2-TZVPPD optimized structures. $CFCl_3$ reference shieldings at the same level have been used to obtain the relative shifts. Nonrelativistic results with pcSseg-4 basis sets for F, Cl, Br, and ANO-RCC-unc for I.

Table S4. Calculated ^{19}F NMR chemical shifts relative to $CFCl_3$ (in ppm) of $[XF_n]^-$ (X = Cl, Br, I; n=2, 4, 6) in comparison with experimental values, at B3LYP-D3 structures.[a]

	BHLYP				LH12ct-SsifPW92				
opt.<br>NMR	gas<br>gas	gas<br>COSMO	COSMO<br>gas	COSMO<br>COSMO	gas<br>gas	gas<br>COSMO	COSMO<br>gas	COSMO<br>COSMO	δ_{exp}
$[ClF_2]^-$	−190.6	−196.4	−193.4	−198.4	−169.8	−171.0	−173.3	−169.8	−125
$[ClF_4]^-$	65.3	61.3	58.5	54.9	56.5	53.8	49.9	47.5	67
$[ClF_6]^-$	250.8	252.9	243.0	245.3	224.7	226.7	217.5	219.7	–
$[BrF_2]^-$	−279.4	−288.5	−281.1	−289.5	−260.0	−267.1	−262.3	−268.9	−210
$[BrF_4]^-$	−38.2	−43.7	−41.5	−46.7	−43.3	−47.8	−46.6	−50.8	−37
$[BrF_6]^-$	113.8	116.4	108.5	111.4	97.6	100.0	92.7	95.3	94
$[IF_2]^-$	−343.0	−351.5	−344.6	−352.9	−332.4	−340.3	−333.9	−341.8	−282
$[IF_4]^-$	−99.5	−105.5	−101.3	−107.1	−107.0	−112.5	−108.8	−114.1	−106
$[IF_6]^-$	29.3	31.1	26.5	28.4	12.7	14.4	9.9	11.7	13
$CFCl_3$	190.77	194.77	189.11	193.25	200.31	203.63	198.72	202.17	

[a] At B3LYP-D3(BJ)/def2-TZVPPD optimized structures. $CFCl_3$ reference shieldings at the same level have been used to obtain the relative shifts. Nonrelativistic results with pcSseg-4 basis sets for F, Cl, Br, and ANO-RCC-unc for I.

Table S5. Fully relativistic 4c-mDKS/B3LYP50/Dyall-VQZ results for ^{19}F NMR nuclear shieldings and chemical shifts of $[XF_n]^-$ (X = Cl, Br, I; n=2, 4, 6).[a]

	$[ClF_2]^-$	$[ClF_4]^-$	$[ClF_6]^-$	$[BrF_2]^-$	$[BrF_4]^-$	$[BrF_6]^-$	$[IF_2]^-$	$[IF_4]^-$	$[IF_6]^-$	$CFCl_3$
σ^{iso}	389.0	126.2	–78.8	471.3	236.1	75.0	539.6	321.5	192.1	192.4
δ	–196.5	66.2	271.2	–278.9	–43.7	117.4	–347.2	–129.2	0.3	0
δ_{exp}	–125	67	—	–210	–37	94	–282	–106	13	0

[a] Gas-phase results relative to $CFCl_3$ at the same level, at BP86-D3(BJ)(COSMO,CH_3CN)/def2-TZVPPD structures.

References

[1] M. Baudler, G. Brauer, *Handbuch der Präparativen Anorganischen Chemie in drei Bänden, Bd1,* Vol 5., Ferdinand Enke, Stuttgart, **1975**, p.166.
[2] R. K. Harris, E. D. Becker, S. M. Cabral de Menezes, P. Granger, R. E. Hoffman, K. W. Zilm, *Pure Appl. Chem.* **2008**, *80*, 59.
[3] G. M. Sheldrick, *Acta Crystallogr. A* **2008**, *A64*, 112.
[4] G. M. Sheldrick, *Acta Crystallogr. C* **2015**, *C71*, 3.
[5] O. V. Dolomanov, L. J. Bourhis, R. J. Gildea, J. A. K. Howard, H. Puschmann, *J. Appl. Cryst.* **2009**, *42*, 339.
[6] K. Brandenburg, *Crystal Impact GbR* **2009**.
[7] Local version derived from Turbomole version 7.4, Turbomole GmbH, 2017. Turbomole is a development of University of Karlsruhe and Forschungszentrum Karlsruhe 1989–2007, Turbomole GmbH since 2007.
[8] a) A. D. Becke *Phys. Rev. A* **1998**, *38*, 3098; b) J. P. Perdew *Phys. Rev. B* **1986**, *33*, 8822.
[9] a) F. Weigend, R. Ahlrichs, *Phys. Chem. Chem. Phys.* **2005**, *7*, 3297; b) D. Rappoport, F. Furche, *J. Chem. Phys.* **2010**, *133*, 134105; c) K. A. Peterson, D. Figgen, E. Goll, H. Stoll, M. Dolg, *J. Chem. Phys.* **2003**, *119*, 11113.
[10] a) S. Grimme, J. Antony, S. Ehrlich, H. Krieg, *J. Chem. Phys.* **2010**, *132*, 154104; b) S. Grimme, S. Ehrlich, L. Goerigk, L. *J. Comput. Chem.* **2011**, *32*, 1456–1465.
[11] A. Klamt, G. Schüürmann, *J. Chem. Soc. Perkin Trans.* **1993**, *2*, 799.
[12] a) A. D. Becke, *J. Chem. Phys.* **1993**, *98*, 5648; b) C. Lee, W. Yang, R. G. Parr, *Phys. Rev. B* **1988**, *37*, 785.
[13] a) F. London, *J. Phys. Radium* **1937**, *8*, 397; b) R. Ditchfield, *Mol. Phys.* **1974**, *27*, 789; c) K. Wolinski, J. F. Hinton, P. Pulay, *J. Am. Chem. Soc.* **1990**, *112*, 8251.
[14] K. Reiter, F. Mack, F. Weigend, *J. Chem. Theory Comput.* **2018**, *14*, 191.
[15] C. J. Schattenberg, K. Reiter, F. Weigend, M. Kaupp *J. Chem. Theory Comput.* **2020**, *16*, 931.
[16] T. Kupka, *Magn. Reson. Chem.* **2009**, *47*, 959.
[17] A. D. Becke, *J. Chem. Phys.* **1993**, *98*, 1372.
[18] A. V. Arbuznikov, M. Kaupp, *J. Chem. Phys.* **2012**, *136*, 014111.
[19] T. M. Maier, A. V. Arbuznikov, M. Kaupp *WIREs Comp. Mol. Sci.* **2019**, *9*, e1378.
[20] F. Jensen, *J. Chem. Theory Comput.* **2015**, *11*, 132.
[21] B. O. Roos, R. Lindh, P.-Å. Malmqvist, V. Veryazov, P.-O. Widmark, *J. Phys. Chem. A* **2005**, *108*, 2851.
[22] S. Komorovský, M. Repiský, O. L. Malkina, V. G. Malkin, I. Malkin Ondík, M. Kaupp *J. Chem. Phys.* **2008**, *128*, 104101/1-15.
[23] M. Repisky, S. Komorovsky, V. G. Malkin, O. L. Malkina, M. Kaupp, K. Ruud, R. Bast, R. Di Remigio, U. Ekstrom, M. Kadek, S. Knecht, L. Konecny, E. Malkin, I. Malkin-Ondik, ReSpect 5.1.0 (2019) Relativistic Spectroscopy DFT program http://www.respectprogram.org.
[24] K. G. Dyall, *Theor. Chem. Acc.* **2006**, *115*, 441.
[25] E. Bernhardt, M. Finze, H. Willner, *J. Fluor. Chem.* **2004**, *125*, 967.
[26] a) X. Zhang, K. Seppelt, *Z. Anorg. Allg. Chem.* **1997**, *623*, 491; b) B. Scheibe, S. I. Ivlev, A. J. Karttunen, F. Kraus, *Eur. J. Inorg. Chem.* **2020**, *37*, 477.
[27] M. J. Turner, J. J. McKinnon, S. K. Wolff, D. J. Grimwood, P. R. Spackman, D. Jayatilaka and M. A. Spackman, *CrystalExplorer17*, University of Western Australia, **2017**.
